# Barriers and Facilitators to the Preadoption of a Computer-Aided Diagnosis Tool for Cervical Cancer: Qualitative Study on Health Care Providers’ Perspectives in Western Cameroon

**DOI:** 10.2196/50124

**Published:** 2025-02-05

**Authors:** Magali Jonnalagedda-Cattin, Alida Manoëla Moukam Datchoua, Virginie Flore Yakam, Bruno Kenfack, Patrick Petignat, Jean-Philippe Thiran, Klaus Schönenberger, Nicole C Schmidt

**Affiliations:** 1 Signal Processing Laboratory LTS5 Swiss Federal Institute of Technology Lausanne (EPFL) Lausanne Switzerland; 2 EssentialTech Centre Swiss Federal Institute of Technology Lausanne (EPFL) Lausanne Switzerland; 3 Department of Obstetrics and Gynaecology Faculty of Medicine and Pharmaceutical Sciences University of Dschang Dschang Cameroon; 4 Institute of Global Health Faculty of Medicine University of Geneva Geneva Switzerland; 5 Department of Obstetrics and Gynecology Annex Regional Hospital of Dschang Dschang Cameroon; 6 Department of Paediatrics, Gynaecology and Obstetrics Faculty of Medicine University of Geneva Geneva Switzerland; 7 Faculty of Social Science Catholic University of Applied Science (KSH) Münich Germany

**Keywords:** qualitative research, technology acceptance, cervical cancer, diagnosis, computer-assisted, decision support systems, artificial intelligence, health personnel attitudes, Cameroon, mobile phone

## Abstract

**Background:**

Computer-aided detection and diagnosis (CAD) systems can enhance the objectivity of visual inspection with acetic acid (VIA), which is widely used in low- and middle-income countries (LMICs) for cervical cancer detection. VIA’s reliance on subjective health care provider (HCP) interpretation introduces variability in diagnostic accuracy. CAD tools can address some limitations; nonetheless, understanding the contextual factors affecting CAD integration is essential for effective adoption and sustained use, particularly in resource-constrained settings.

**Objective:**

This study investigated the barriers and facilitators perceived by HCPs in Western Cameroon regarding sustained CAD tool use for cervical cancer detection using VIA. The aim was to guide smooth technology adoption in similar settings by identifying specific barriers and facilitators and optimizing CAD’s potential benefits while minimizing obstacles.

**Methods:**

The perspectives of HCPs on adopting CAD for VIA were explored using a qualitative methodology. The study participants included 8 HCPs (6 midwives and 2 gynecologists) working in the Dschang district, Cameroon. Focus group discussions were conducted with midwives, while individual interviews were conducted with gynecologists to comprehend unique perspectives. Each interview was audio-recorded, transcribed, and independently coded by 2 researchers using the ATLAS.ti (Lumivero, LLC) software. The technology acceptance lifecycle framework guided the content analysis, focusing on the preadoption phases to examine the perceived acceptability and initial acceptance of the CAD tool in clinical workflows. The study findings were reported adhering to the COREQ (Consolidated Criteria for Reporting Qualitative Research) and SRQR (Standards for Reporting Qualitative Research) checklists.

**Results:**

Key elements influencing the sustained use of CAD tools for VIA by HCPs were identified, primarily within the technology acceptance lifecycle’s preadoption framework. Barriers included the system’s ease of use, particularly challenges associated with image acquisition, concerns over confidentiality and data security, limited infrastructure and resources such as the internet and device quality, and potential workflow changes. Facilitators encompassed the perceived improved patient care, the potential for enhanced diagnostic accuracy, and the integration of CAD tools into routine clinical practices, provided that infrastructure and training were adequate. The HCPs emphasized the importance of clinical validation, usability testing, and iterative feedback mechanisms to build trust in the CAD tool’s accuracy and utility.

**Conclusions:**

This study provides practical insights from HCPs in Western Cameroon regarding the adoption of CAD tools for VIA in clinical settings. CAD technology can aid diagnostic objectivity; however, data management, workflow adaptation, and infrastructure limitations must be addressed to avoid “pilotitis”—the failure of digital health tools to progress beyond the pilot phase. Effective implementation requires comprehensive technology management, including regulatory compliance, infrastructure support, and user-focused training. Involving end users can ensure that CAD tools are fully integrated and embraced in LMICs to aid cervical cancer screening.

## Introduction

### Background

In low- and middle-income countries (LMIC), visual inspection with acetic acid (VIA) is a common low-cost method for screening and triage. This method involves applying diluted acetic acid to the cervix during gynecological examination. This induces tissue whitening, which a trained observer assesses to guide the diagnosis. Despite VIA’s cost-effectiveness and accessibility, a major limitation is its high subjectivity owing to variability in the training and experience of the observer [1].

With advancements in artificial intelligence (AI), its potential to assist in diagnosis has been extensively explored and investigated [2]. Tools that aid health care providers (HCPs) in detecting diseases and identifying abnormalities are clinical decision support (CDS) systems and, more specifically, computer-aided detection and diagnosis (CAD) systems [3]. In cervical cancer, CAD tools can mitigate the subjectivity inherent in VIA by providing standardized evidence for clinical decision-making [4].

Despite the promising potential of CAD systems, their implementation and sustained use in LMICs are limited, irrespective of the target disease. These barriers include the risk of workflow disruption, dependency on computer literacy, poor data quality, lack of transportability and interoperability (ie, system compatibility), and financial challenges [5]. In addition, frameworks exist to guide the implementation, evaluation, and regulation of these digital health tools [6-10]. However, the lack of harmonization across different entities compounds their development complexity. The technology may not progress beyond the pilot stage because of the previously mentioned reasons—a common phenomenon referred to as “pilotitis” [11].

This study examines the barriers and facilitators to deploying a VIA CAD tool to enhance and standardize cervical cancer diagnosis using AI. User perspectives were collected to understand the challenges and enablers of implementing this technology. This study provides insights into deploying AI-enhanced diagnostic tools in LMICs to bridge the gap between technological development and real-world implementation.

### Theoretical Framework

In the context of VIA CAD tools for LMICs, the following definitions are used throughout the paper: (1) acceptability: “the quality of being satisfactory and able to be agreed to or approved of,” according to the Cambridge Dictionary [12]; (2) acceptance: “general agreement that something is satisfactory or right,” as stated by the Cambridge Dictionary [13]; and (3) adoption: “a multiphase process starting with deciding to adopt [a technology] (selecting, purchasing, or committing to use it) and then achieving persistent use,” provided by Carroll et al [14].

**Figure 1:**
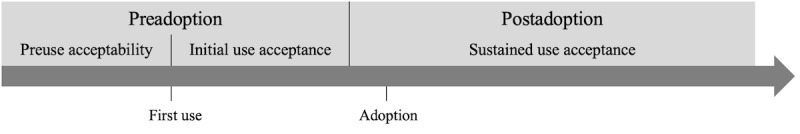
Technology acceptance lifecycle adapted from Nadal et al [15].

These 3 concepts are combined in successive steps in the technology acceptance lifecycle (TAL) proposed by Nadal et al [15]. The model (Figure 1) reveals that technology preadoption is a 2-stage process. First, acceptability before use is preuse acceptability, followed by initial use acceptance once the technology has been used for the first time. Postadoption is considered sustained use acceptance, implying that the device is fully adopted and sustainably used.

The TAL model was chosen because it captures key factors in the preadoption phase, which is crucial previous to integrating CAD tools in resource-constrained health care settings. Furthermore, the postadoption phase addresses specific barriers, such as infrastructure and data security, while simultaneously supporting sustained adoption and mitigating pilotitis risk.

### Objective

This study focused on the challenges perceived by HCPs that would prevent the sustained use of CAD tools for VIA in clinical settings. The primary objective of this study was to identify the common barriers to adopting CAD tools for VIA, and facilitators were the secondary objective. Barriers and facilitators were studied within the framework of the TAL, especially in the preadoption phase. The study was conducted in Western Cameroon; however, the results are critically interpreted for generalizability to other geographical settings within sub-Saharan Africa or other LMICs.

## Methods

### Overview

This study is reported per the COREQ (Consolidated Criteria for Reporting Qualitative Research; Multimedia Appendix 1) and SRQR (Standards for Reporting Qualitative Research; Multimedia Appendix 2) checklists.

### Study Settings and Recruitment

This study was conducted as part of a cervical cancer screening program in the Dschang district, Western Cameroon. A larger study, the 3T program (for test-triage-treatment), was initiated in 2018 at Dschang District Hospital with the support of the Ministry of Health and in collaboration with Geneva University Hospital [16]. This program includes patient recruitment through awareness campaigns in rural and urban areas, tests for HPV, followed by VIA triage (if the HPV test is positive), and treatment, if necessary. The cervical cancer screening was performed by midwives who welcomed the patients, explained the study, conducted the gynecological examination, and, if required, administered treatment. In the case of severe lesions, the patient was treated by a midwife and gynecologist. Within the scope of this program, images were collected during the colposcopy for peer review of the diagnoses and to develop a CAD tool for VIA. This technology relies on image processing and machine learning [17].

Participants were recruited from trained teams at the Dschang District Hospital and the Regional Hospital of Bafoussam, a second 3T program site approximately 60 km east of Dschang. Every HCP in the 3T program is Cameroonian, better to understand the local context and its specific culture. Participants were compensated for their transportation expenses and provided with refreshments during discussions.

### Study Design and Procedure

A qualitative methodology was chosen to capture nuances in participants’ views, experiences, and behaviors regarding technology and their environment [18]. Compared with a quantitative approach, a qualitative approach provides better insight into participants’ perspectives and provides the flexibility required for participants to highlight information they may not have anticipated [19,20].

A mixed approach composed of individual interviews and mini–focus group discussions (mFGDs) was adopted. Individual interviews contributed to in-depth data collection [21], and mFGDs facilitated the exchange of participants’ perspectives and the compilation of collective perceptions [20]. The term “mini” denotes the small group size of 3 participants, which was chosen because of the limited availability of individuals with relevant expertise and the sensitivity of the topic, which benefits from an intimate setting [22]. Due to the HCPs’ various educational levels, training, and daily duties, the participants were grouped into homogeneous professional mFGDs. The aim was to create a climate of trust, enabling all participants to express themselves freely and reduce authority bias [23].

Therefore, 2 mFGDs, each with 3 midwives and 3 individual interviews with gynecologists, were conducted between March and May 2022. All discussions were moderated in French—the official language—by a female Cameroonian anthropologist who had worked on the 3T program for several years and had intimate knowledge and familiarity with the professional community. During focus group discussions, in which the perspectives of the HCPs were collected at the Dschang District Hospital, a second female Cameroonian anthropologist took notes to capture nonverbal cues and additional context. An individual interview was conducted via videoconferencing because the medical doctor was traveling. The remaining discussions occurred in clinical settings, either in a confidential conference room at the hospital or a private medical office.

Each interview began with an introduction to the CAD tools for VIA, and the procedure was described in detail. The slides supported the verbal explanation and clarified the following steps. During the gynecological examination, the HCP first applied diluted acetic acid to the cervix, corresponding to the routine VIA. Second, cervical images were recorded using a dedicated mobile app. Subsequently, the algorithm integrated into the mobile app processed the sequence of images and provided an analysis to the user. Finally, the HCP interpreted the results to determine whether treatment was necessary. The entire procedure can be conducted offline; nonetheless, internet-based synchronization with a server is also feasible, ensuring data backup and compliance with data privacy and confidentiality requirements.

A mobile app was specifically developed for the demonstration to concretize the concept for the participants. The mobile app allowed users to capture a series of pictures and generate simulated predictions of cervical cancer. The participants could manipulate the mobile app and take pictures of their surroundings to familiarize themselves with CAD tools for cervical cancer. A semistructured questionnaire was then used to (1) investigate HCPs’ perceptions of smartphone use for medical applications, cervical cancer, and AI and (2) identify the challenges and facilitators for integrating CAD tools for VIA in clinical settings.

All interview guide questions were fully addressed, and the participants were given opportunities to ask questions. Finally, participants could share their thoughts and comments. All discussions were audio-recorded, transcribed, and anonymized. Triangulation was applied within and across sessions to enhance data reliability.

### Data Processing and Analysis

A total of 4 audio recordings were transcribed verbatim in French, categorized, and coded using content analysis [24]. Therefore, coding was initially deductive based on a codebook, guided by the topics outlined in the interview guide, followed by the generation of inductive codes directly from the transcriptions. Around 2 female coresearchers (MJC and AMDM) independently coded transcripts using the computer-assisted qualitative data analysis software (ATLAS.ti, version 22.2.3) to support scientific rigor and reflexivity. The data were double-coded by a researcher from a different cultural context to address the potential reporting bias that could arise from the shared experience between the anthropologist and the HCPs. The coding was then compared, discussed when diverging, combined, and finally assessed for consistency following the open discussion method [25].

The coded data were analyzed to identify relationships between the categories and hierarchically ordered, with the TAL framework applied as a theoretical lens for interpreting the coded data. Data were analyzed with team members from diverse professional and cultural backgrounds. This combination of expertise and cultural insights strengthened the study design and analysis. Data saturation was confirmed through consistent responses across the mFGDs and interviews. Each mFGD was included, and all sessions were audio-recorded while maintaining confidentiality. Suppose quotations were selected for publication; in that case, they were translated from French into English.

### Ethical Considerations

This study was approved by the Ethical Cantonal Board of Geneva, Switzerland (CCER, 2017-01110 and CER-amendment no. 4) and the Cameroonian National Ethics Committee for Human Health Research (2022/12/1518/CE/CNERSH/SP). The participants verbally consented after being informed of the study’s purpose, topics, duration, benefits, and risks. Participants were compensated for their transportation expenses. All transcripts were anonymized and analyzed in a fully deidentified manner to ensure privacy and confidentiality. The achievement of data saturation, the presence of 2 researchers during the mFGDs, audio recordings, and triangulation of information attest to the rigor and trustworthiness of the data collection and analysis.

## Results

### Participants

The study involved all HCPs from both 3T program sites, encompassing 6 midwives and 2 gynecologists, of whom 5 were females and 3 were male participants aged 30-55 years. All participants specialized in cervical cancer, with experience between 6 months and over 10 years, providing insights for evaluating the technology across different stages of professional development. They all owned a smartphone, used it daily, and were familiar with smartphone apps in clinical settings through the 3T program. None of the participants dropped out of the study, and the discussion duration was 42-92 minutes.

### Main Results

HCPs’ perceptions of adopting a CAD tool for VIA were mainly positive. The HCP highlighted 8 facilitators and 5 barriers that should be addressed in the future.

The barriers were:

- Restriction of the HCP’s movement due to the smartphone’s position.
- Constraining requirements for the image quality.
- Confidentiality concerns for sensitive data, especially in case of data breach.
- Workflow changes may encourage HCPs to heavily depend on the technology.
- Limited access to internet connection and smartphones.

The facilitators were:

- Improved patient care through rapid and reliable diagnosis.
- Improved diagnosis.
- Reduced workload because of improved efficiency.
- Reinforcement of visual inspection with acetic acid (VIA) protocol.
- Clinical evaluation of the technology in conditions reflecting real-life conditions.
- Automated assessment of image quality.
- Training of the users.
- Provision of smartphones and tripods.

### Barriers to CAD Tools for VIA

#### System Usability Challenges

The smartphone was positioned on a tripod between the gynecological chair and HCP to capture the images. This positioning restricted the movement of professionals during the examination.

When pipetting [acetic acid] and the whole cervix is not captured on the smartphone but the recording already started, then we need to twist and turn to finish. For those difficulties, we could pull the trip away, pipet to remove all the acetic acid well, and then position the device.

P6

In addition, image quality requirements entail acquisition conditions that are difficult to achieve. These quality assessment criteria can be affected by video movements, light changes, reflections, and the presence of blood or mucus. Furthermore, some HCPs raised concerns that the high-quality images required for an accurate diagnosis would be difficult to achieve, possibly leading to misdiagnosis.

[…] So that artificial intelligence provides an accurate result, one will need to fulfill all acquisition conditions. It means that if you make a small mistake in the process, the risk of a false positive or false negative will be high […]

P2

#### Confidentiality

Most HCPs expressed apprehension regarding the handling and storage of sensitive data. Their concerns revolved around potential breaches of medical data confidentiality and the unauthorized sharing of VIA images. Furthermore, they feared the loss of images due to the misfunctioning or mishandling of smartphones.

[…] If I send a picture to someone, they can easily forward it to someone else without asking my permission. There were so many scandals in the field of medicine because someone took a picture with their smartphone, which was shared multiple times and leaked […]

P8

An HCP emphasized that framing the picture around the cervix alone can aid confidentiality concerns because it is anonymizing.

When using a smartphone to take photos of the cervix, it is beneficial as long as it preserves the woman’s privacy by capturing solely the cervix. That way, the woman cannot feel frustrated about her face potentially being seen elsewhere.

P2

In addition, using a private mobile phone to take pictures during the gynecological examination was not allowed. The device should be dedicated to only clinical settings and not leave the screening area.

[…] the personal mobile phone is never fully private… when you drop it at home, kids may search into it and access the image […] Personally, I believe the mobile phone used needs to be professional only and to stay at the screening site, never reaching someone’s home […]

P8

#### Workflow Changes

Several HCPs were concerned that the adaptation of the device might change the workflow, and users might heavily depend on CAD tools, neglecting their expertise. An HCP explained:

For me, and my colleagues, if this device is given to medical doctors, they might become lazy. They will not have to do their work fully. As soon as there is a case, they would take the smartphone and would not think about the diagnosis themselves.

P1

Therefore, their gynecological knowledge might be affected, possibly limiting patient care in complicated cases.

#### Limited Infrastructure and Resources

Another challenge regarding adopting the technology is the limited access to resources. Some HCPs were concerned about access to reliable internet connections and smartphones, along with their quality. The wear of the smartphone battery was also a concern.

[…] Everyone cannot access a smartphone, everyone cannot access the internet […]

P8

### Facilitators of CAD Tools for VIA

#### Patient Care Improvement

Most HCPs agreed that the technology would improve patient care by providing a rapid and reliable diagnosis. According to them, accelerating the diagnosis speed benefited the patients and HCPs. First, patients would not return home with unanswered questions and directives to wait, which could induce stress and unnecessary worry.

They [patients] will accept the technology since when coming out of here [the hospital], they will know their diagnosis; they will think, ‘They told me that I come back home with a clear head, not like before when I came and I had to wait for a phone call with the results […]

P3

Second, the technology could help HCPs provide a diagnosis or mitigate the lack of resources for conducting biopsies. Some professionals also believed that the workload could be reduced using technology that would enable the screening of more women for cervical cancer.

Furthermore, some HCPs mentioned that adopting a CAD tool for cervical cancer could enhance diagnosis by identifying lesions that are challenging to detect with the human eye. This can reduce the potential for human error.

If it is efficient, then it would be a very important tool because visual inspection is now very subjective. But if we manage to generate a diagnosis from a simple image, that would be a very good progress.

P7

This technology may also reinforce the protocol for performing appropriate VIA. For example, the predetermined duration of the recording constrains the user to wait until the recording is completed. Diagnosis improvements may also reduce the rate of overtreatment, which is currently considered crucial, as illustrated by one of the HCPs.

[…] in the approach to screen and treat, we are afraid, on the one hand, not to treat someone who should have, until the point it develops into a cancer. On the other hand, we may treat someone who did not need it, which is called overtreatment, but if we have this technology helping, then we will win in both cases […]

P7

#### Clinical Evaluation

Before this technology can be used in clinical settings, approval and validation by health care professionals is required. This implies comparing the technology’s diagnosis in real conditions and that of HCPs, histopathology results, and a usability study regarding the tool’s features and interface. The HCPs confirmed the need to validate the technology because they were concerned about its performance and their ability to distinguish accurate diagnoses from misdiagnoses.

#### Automated Assessment of Image Quality

Some HCPs apprehend that automating the technology would induce misdiagnosis and false-positive and false-negative predictions because of cervical abnormalities such as blood, mucus, and benign lesions. Therefore, an automated pipeline for assessing image quality was indicated as an essential technological feature preventing misdiagnosis by not allowing the application of the algorithm to images that are of extremely low quality or ineligible.

For instance, we could have a feature that gives us an ok when all acquisition criteria are fulfilled and that the analysis of the images can be conducted. Or, if criteria are not fulfilled, then there would be a message telling you not to launch the analysis and to, maybe, retake images.

P4

#### Training and Resources Provision

The participants highlighted the importance of comprehensive training on properly using the CAD tool for its clinical adoption. In addition, resources such as smartphones and tripods are required for effective implementation.

[…] One needs to ensure that the user of the technology is well trained to create conditions allowing the capture of a good image because… bad acquisition conditions result in bad image quality.

P7

## Discussion

### Principal Findings

This study identified 4 barriers and 4 facilitators each to adopting CAD tools for VIA during the preadaptation phase of the TAL model (Figure 1), either related to preuse acceptability or to initial use acceptance (Table 1). These included structural components such as limited infrastructure, confidentiality, data management, and personal factors such as workflow changes and user interaction with the CAD tool. HCPs emphasized the overall benefits, including improved patient care through better clinical evaluation, enhanced training, and technical advantages, such as ease of use of the system. The following section discusses the barriers and facilitators identified in both phases, contextualized within this literature, and highlights the barriers observed in previous studies but not encountered in this one.

**Table 1:** Synthesis of principal findings under the preadoption phases of the technology acceptance life cycle.

	Preuse acceptability	Initial use acceptance
**Barriers**	Confidentiality and data management	User interaction
	Limited infrastructure and resources	Change in workflow
**Facilitators**	Patient care improvement	Clinical evaluation
	Training	Ease of use

### Barriers to Preuse Acceptability

#### Confidentiality and Data Management

Confidentiality concerns were highlighted as a significant factor affecting pre-use acceptability. HCPs expressed reservations about data loss and breaches and the acquisition of cervical images with smartphones. Patients also expressed concerns about the image frame. However, these concerns are not specific to the use of AI but are raised by the use of digital, sensitive images. Lodhia et al [26] observed that assuring patients of confidentiality was crucial to their mHealth intervention study for eye care in Kenya and recommended a robust data protection system. Prioritizing confidentiality, incorporating a secure data management system, and transparent communication about security measures are essential for alleviating privacy concerns and building trust among HCPs and patients. Practically, some HCPs have suggested showing the acquired data to patients to reassure them about the content of the images.

#### Limited Infrastructure and Resources

A lack of infrastructure and resources can affect the preuse acceptability of CAD tools for VIA in cervical cancer. Equipping HCPs with the necessary devices (such as smartphones and tripods) and ensuring regular maintenance of the devices is essential for limiting technical challenges and providing uninterrupted and effective health care services to patients.

Limited access to reliable internet connections, smartphone quality, and smartphone battery wear concerns during prolonged use have been highlighted as barriers by HCPs. These challenges align with findings from the existing literature in which infrastructure-related challenges have been identified in other health care settings. A study by Elahi et al [27] on a CAD tool for traumatic brain injury in Uganda emphasized the importance of internet connection and the associated costs. Spence et al [28] and Knoble and Bhusal [29] reported reliable electricity as a key challenge in the studies of childhood pneumonia diagnostic tools and electronic diagnostic algorithms.

A viable solution to overcome internet-related constraints is developing a CAD tool that can function offline. Internet access might be occasionally needed for maintenance, updates, and data sharing or backup; however, the device would provide CDS, irrespective of internet coverage and connectivity. In addition, ensuring access to electricity is essential to keep the smartphones charged. Therefore, adopting good practices, such as switching off the device at night or when it is not in use for several days, can help preserve battery life. Furthermore, optimizing the CDS algorithm to minimize smartphone power consumption can contribute to its ease of use without frequent recharging. By implementing these strategies, HCPs can confidently use CAD tools, even in remote areas with limited internet access and electricity.

### Facilitators for Preuse Acceptability

#### Patient Care Improvement

CAD tools for VIA have diverse benefits that may contribute to improvements in cervical cancer screening and diagnosis [30]. A key advantage is rapid and reliable diagnosis, which reduces waiting time for patients who can receive test results and treatment in a single appointment. This point-of-care approach can enhance patient satisfaction and positively impact mental health by providing a timely, near–real-time diagnosis [31]. In addition, this technology serves as a valuable supplementary test, assisting HCPs and making the diagnostic process less subjective. This technology limits cases of overtreatment and mitigates the risk of unnecessary treatment-related complications, such as pregnancy-related morbidity, by enhancing diagnosis accuracy [32].

#### Training

Comprehensive training is essential for users to understand and use the technology accurately. HCPs should not rely solely on the suggested diagnosis but use it as an assistive device that provides supplementary evidence to be considered in addition to their clinical knowledge and experience. Training must be tailored to the specific contexts of use and encompass geographical, cultural, and educational considerations [33]. The objective is to equip users with the ability to comprehend, interpret, and integrate recommendations from CAD tools into their decision-making processes.

### Barriers to Initial Use Acceptance

#### User Interaction

The initial acceptance of the system can be hindered by difficulty in use. Demanding acquisition conditions are challenging for HCPs to comply with, potentially hindering technology adoption. The CDS algorithm requires adherence to specific acquisition conditions to address issues, such as movement, light, and mucus or blood in the cervix. The HCPs suggest that this could be facilitated by an automated quality assessment pipeline that assesses image quality in real time and provides immediate feedback to users, indicating whether the image quality is sufficient. Such a process could assess the visibility of the cervix, monitor movement, detect blurriness, or detect external objects (eg, pipettes).

Beyond the acquisition process, interaction with the device must be satisfactory to the HCPs. Knoble [29] conducted a comprehensive study on electronic diagnostic algorithms in Nepal and uncovered that HCPs found the device’s size too small and the touchscreen sensitivity too low. This highlights the importance of considering all usability aspects while developing the technology and iteratively testing it with users.

#### Workflow Changes

Potential changes in the HCPs workflow also need to be assessed because they could hinder the initial acceptance of CAD tools for VIA [34]. Furthermore, a few HCPs expressed concerns about the risk of neglecting their expertise because of heavy reliance on the new technology. This observation is consistent with current literature. Despite the potential educational purposes of AI-driven CDS tools, the tendency to rely heavily on automation, described as automation bias, has been highlighted by Khera et al [35]. Furthermore, Jabbour et al [36] observed a decline in diagnostic performance when clinicians used AI support, even when visual explanations of AI-driven diagnoses were provided.

Introducing a novel technology can also increase the workload during the initial learning phase. Melas et al [37] observed that reduced time consumption was a vital factor influencing clinicians’ intentions to adopt it. In contrast, Ellington et al [38] reported that HCPs need to practice using technology before using it with patients to improve efficiency. This preparatory practice would allow HCPs to become familiarized with the technology’s functionalities and workflows, ensuring a smoother and more efficient integration of the technology into their clinical practice.

### Facilitators for Initial Use Acceptance

#### Clinical Evaluation

The technology should undergo rigorous evaluation to assess its clinical and cost benefits to ensure initial use acceptance and foster sustained use acceptance [9]. The tool’s performance can be evaluated using metrics such as sensitivity, specificity, accuracy, and false-positive rate. Evaluating the efficiency of the device and its integration into patient care contributes to assessing its clinical benefits. This involves examining the clinical utility of the technology, its compatibility with specific clinical settings, and its impact on patient outcomes. In addition, a user-centered design highlights the importance of user feedback for validating the technology, particularly its interface, features, and ease of use [39]. Cost benefits should also be assessed by estimating cost savings and implementation costs, including initial setup, training, and maintenance costs.

Laka et al [40] highlighted that evaluation frameworks should consider the dynamic nature of clinical settings and CDS (eg, through software updates). However, such devices need to be continuously monitored to ensure their safe and effective integration in clinical settings [41]. Papadopoulos et al [42] illustrated this challenge in a systematic literature review, revealing that only a few studies included “any form of evaluation,” with often insufficient methodologies.

#### Ease of Use

In addition to facilitating the acquisition and quality assessment processes, simplicity in using CAD tools is crucial for their successful adoption in clinical settings [28]. Panicker et al [43] indicated that easy-to-use CAD tools save time because of their simple operation, fast access, effective recording, and information retrieval. These features contribute to the perceived usefulness and potential for extensive system adoption in clinical practice.

### Comparison to Previous Studies

The barriers identified mostly align with those in the existing literature. However, these studies also reported barriers not mentioned by the participants of this study.

A common barrier highlighted in the literature is the limited resources to finance the technology, cope with increased diagnosis, and provide follow-up and treatment when necessary [26-28,44]. In the 3T program, external funding covers all costs, including salaries, clinical materials (gloves, speculum, acetic acid, etc), equipment (smartphone, tripod, etc), and patient travel cost compensation, eliminating immediate financial constraints.

Another barrier emerging from the literature, but not specifically addressed in this study, is the risk of mobile device theft [26]. This could be a relevant consideration in less controlled settings. Community and political support are also lacking in the literature [38,43]. The 3T program receives support from the Health Ministry, albeit financed through foreign grants.

The literature indicates a lack of user experience or skill (ie, phone literacy) as a potential barrier, even though the participants were comfortable handling smartphones [26,29,38,43]. This may result in misuse or an increased workload. In the 3T program, all HCPs follow comprehensive training, ensuring their proficiency in using the technology.

In summary, the absence of these barriers identified in the international scientific literature but not encountered in the 3T program might be attributed to the specific context and controlled environment provided by the program, as well as the active involvement of the study participants in it.

### Further Recommendations

The World Health Organization (WHO) proposes 3 main solutions for barriers to the general use of medical devices [45]. First, the technology should be designed to fit the context of use. Second, the device system should be managed comprehensively, including its regulatory aspects, installation, maintenance, and monitoring. Finally, professionals need to be trained for proper use, maintenance, and presentation to patients. In alignment with the WHO recommendations to avoid pilotitis, this study identified barriers and facilitators to integrating CAD tools for VIA in cervical cancer diagnosis.

Addressing specific barriers to deploying CAD systems involves the fundamental element of trust. Patients and HCPs must trust the technology to ensure its successful implementation and sustained use. Trust might motivate HCPs to integrate CAD systems into their workflows and enhance patients’ acceptance and comfort through a diagnostic process facilitated by CAD tools.

Trust can be built by involving users from the outset of technological development and integrating their feedback. Early engagement allows the understanding of their specific needs and ensures that the technology addresses relevant challenges. Involving various user profiles (eg, profession, degree, and working experience) from different clinical settings introduces a range of perspectives that can be leveraged to tailor the technology to diverse contexts.

In addition, providing evidence of the technology’s trustworthiness is key to building trust [46]. Explainability, which makes the decision-making process of the technology transparent and understandable to users, builds confidence in its reliability. Traditionally, CDS are knowledge-based and rely on medical literature and conditional logic [47]. This approach tends to be more transparent than the current AI-leveraged CDS systems. Comprehensive training further reinforces trust for knowledge- and AI-based CDS by ensuring the appropriate use of the technology through stepwise instructions and presentation of its limitations.

The TAL framework provides valuable perspectives on the current stages of technological adoption. However, it should be considered as an iterative tool, acknowledging that perceptions and acceptance may evolve as the technology progresses. Continuous monitoring of these changes is essential to ensure the successful adoption and use of CAD tools for VIA in clinical practice.

### Strengths and Limitations

To our knowledge, this is one of the first studies in sub-Saharan Africa that investigates the adoption of CAD tools for cervical cancer from the perspective of HCPs. The various professional backgrounds of the participants, years of experience in cervical cancer screening, and sex allowed us to gain in-depth insights into the barriers and facilitators present during the preadaptation phase.

The qualitative method allowed for collecting different perspectives; however, several limitations were observed. First, the study involved a limited number of nonrandom samples of participants familiar with the use of smartphones for cervical cancer diagnosis. In addition to potential selection bias and limited generalizability, mini–focus groups have been acknowledged in the scientific literature, especially among participants with intense experience with a topic, which was the case in our study, allowing for a more in-depth discussion [48]. The mFGDs were complemented by individual interviews to capture a wider range of perspectives and mitigate this bias. Second, the study was conducted in a controlled program in Western Cameroon, which may not reflect other settings with varying resources and infrastructure. Therefore, we recommend replicating the study in other contexts for broader validation. Third, this study focused on HCPs; however, patient perspectives, crucial for understanding technology adoption, were not included but addressed in a separate study [49]. Finally, barriers can be attributed to the use of CAD tools or smartphones. This study was conducted during the preadaptation phase; however, the technology and user attitudes have evolved. Iterative research is needed to determine whether factors can be attributed to specific digital tools and capture changing perceptions after implementing the CAD tool.

Some of the encountered barriers (such as workflow changes) might be addressed through adaptation and facilitator reinforcement (such as training); therefore, the findings of this study provide a crucial foundation for future work. In summary, further qualitative and quantitative studies should be conducted among HCPs with less smartphone experience and in various clinical settings to enhance the generalizability of findings and guide tailored strategies for implementing CAD tools in cervical cancer diagnosis.

### Conclusions

Interviewing HCPs from various backgrounds with diverse working experience—midwives and gynecologists—was essential to better understand their concerns, perceptions, and expectations regarding a CAD tool for cervical cancer and to avoid “pilotitis.”

Even if a CAD tool is designed to fit its context of use perfectly, understanding the barriers and facilitators is key to its successful implementation and use. In this study, the CAD tool was appreciated by the HCP for its ease of use and crucial role as an assistive device to improve patient care and support clinical decisions. The barriers encountered (confidentiality and data management, limited infrastructure and resources, workflow changes, and user interaction) are specific to the study setting and might vary with the context. However, these challenges can be addressed in line with the WHO recommendations [40] by ensuring proper management of the technology (eg, regulation and maintenance) and involving the end user at every step of developing the solution. HCPs will help define the appropriate way of introducing new technology to patients and their peers, as well as how to train them for proper use. These measures aim to derisk the deployment of the technology and contribute to overcoming pilotitis.

## Appendix

Multimedia Appendix 1 Consolidated criteria for reporting qualitative research (COREQ) checklist.

Multimedia Appendix 2 Standards for Reporting Qualitative Research (SRQR) checklist.

## Abbreviations

AI: artificial intelligence
CAD: computer-aided diagnosis CDS: clinical decision support
COREQ: Consolidated Criteria for Reporting Qualitative Research HCP: health care provider LMIC: low- and middle-income country mFGD: mini–focus group discussion
SRQR: Standards for Reporting Qualitative Research TAL: technology acceptance lifecycle VIA: visual inspection with acetic acid WHO: World Health Organization

## Data Availability

The datasets generated and analyzed during this study are not publicly available due to confidentiality and privacy concerns but from the corresponding author on reasonable request.
