# Supplementary material for: Barriers and Facilitators to the Preadoption of a Computer-Aided Diagnosis Tool for Cervical Cancer: Qualitative Study on Health Care Providers’ Perspectives in Western Cameroon
Source: JMIR Cancer. 2025 Feb 5;11:e50124. doi: 10.2196/50124 (PMC11840362; doi:10.2196/50124)
Supplement: Multimedia Appendix 1 [file cancer_v11i1e50124_app1.docx]

| Multimedia Appendix: Consolidated criteria for reporting qualitative research (COREQ) checklist | | | |
| --- | --- | --- | --- |
| <https://www.equator-network.org/reporting-guidelines/coreq/>  Checklist from ﻿Tong, A., Sainsbury, P., & Craig, J. (2007). Consolidated criteria for reporting qualitative research (COREQ): a 32-item checklist for interviews and focus groups. International Journal for Quality in Health Care, 19(6), 349–357. [https://doi.org/10.1093/INTQHC/MZM042](file:////Users/nicoleschmidt/Desktop/KSH%20/Forschung/2024/Magali_Mansucript/V3.12.2024/1.%20Tong,%20A.,%20Sainsbury,%20P.,%20&%20Craig,%20J.%20(2007).%20Consolidated%20criteria%20for%20reporting%20qualitative%20research%20(COREQ):%20a%2032-item%20checklist%20for%20interviews%20and%20focus%20groups.%20International%20Journal%20for%20Quality%20in%20Health%20Care,%2019(6),%20349–357.%20https:/doi.org/10.1093/INTQHC/MZM042)  Completed in relation to ﻿Jonnalagedda-Cattin, M., Moukam Datchoua, A., Yakam, V. F., Kenfack, B., Petignat, P., Thiran, J.-P., Schönenberg, K., Schmidt, N. C. Barriers and facilitators for the pre-adoption of computer-aided diagnosis tools for cervical cancer: A qualitative study on healthcare providers’ perspectives in Western Cameroon. | | | |
|  |  |  | **Page no(s).** |
| **Domain 1: Research team and reﬂexivity** | | |  |
| *Personal Characteristics* | | |  |
|  | 1. Interviewer/facilitator | Which author/s conducted the interview or focus group? | p. 14 |
|  | 2. Credentials | What were the researcher’s credentials? E.g. PhD, MD | p. 1 |
|  | 3. Occupation | What was their occupation at the time of the study? | p. 5 |
|  | 4. Gender | Was the researcher male or female? | p. 5 |
|  | 5. Experience and training | What experience or training did the researcher have? | p. 5 |
| *Relationship with participants* | | |  |
|  | 6. Relationship established | Was a relationship established prior to study commencement? | p. 4 |
|  | 7. Participant knowledge of the interviewer | What did the participants know about the researcher? e.g. personal goals, reasons for doing the research | p. 4 |
|  | 8. Interviewer characteristics | What characteristics were reported about the interviewer/facilitator? e.g. Bias, assumptions, reasons and interests in the research topic | p. 4 |
| **Domain 2: study design** | | |  |
| *Theoretical framework* | | |  |
|  | 9. Methodological orientation and Theory | What methodological orientation was stated to underpin the study? e.g. grounded theory, discourse analysis, ethnography, phenomenology, content analysis | p.3, p. 5 |
| *Participant selection* | | |  |
|  | 10. Sampling | How were participants selected? e.g. purposive, convenience, consecutive, snowball | p. 4 |
|  | 11. Method of approach | How were participants approached? e.g. face-to-face, telephone, mail, email | p. 4 |
|  | 12. Sample size | How many participants were in the study? | p. 6 |
|  | 13. Non-participation | How many people refused to participate or dropped out? Reasons? | p. 6 |
| *Setting* | | |  |
|  | 14. Setting of data collection | Where was the data collected? e.g. home, clinic, workplace | p. 4 |
|  | 15. Presence of non-participants | Was anyone else present besides the participants and researchers? | p. 4 |
|  | 16. Description of sample | What are the important characteristics of the sample? e.g. demographic data, date | p. 6 |
| *Data collection* | |  |  |
|  | 17. Interview guide | Were questions, prompts, guides provided by the authors? Was it pilot tested? | p. 4 |
|  | 18. Repeat interviews | Were repeat interviews carried out? If yes, how many? | N/A |
|  | 19. Audio/visual recording | Did the research use audio or visual recording to collect the data? | p. 5 |
|  | 20. Field notes | Were ﬁeld notes made during and/or after the interview or focus group? | p. 4 |
|  | 21. Duration | What was the duration of the interviews or focus group? | p. 6 |
|  | 22. Data saturation | Was data saturation discussed? | p. 5 |
|  | 23. Transcripts returned | Were transcripts returned to participants for comment and/or correction? | N/A |
| **Domain 3: analysis and ﬁndings** | | |  |
| *Data analysis* | |  |  |
|  | 24. Number of data coders | How many data coders coded the data? | p. 5 |
|  | 25. Description of the coding tree | Did authors provide a description of the coding tree? | N/A |
|  | 26. Derivation of themes | Were themes identiﬁed in advance or derived from the data? | p. 5 |
|  | 27. Software | What software, if applicable, was used to manage the data? | p. 5 |
|  | 28. Participant checking | Did participants provide feedback on the ﬁndings? | N/A |
| *Reporting* | |  |  |
|  | 29. Quotations presented | Were participant quotations presented to illustrate the themes/ﬁndings? Was each quotation identiﬁed? e.g. participant number | pp. 6-9 |
|  | 30. Data and ﬁndings consistent | Was there consistency between the data presented and the ﬁndings? | p. 13 |
|  | 31. Clarity of major themes | Were major themes clearly presented in the ﬁndings? | pp. 8-9 |
|  | 32. Clarity of minor themes | Is there a description of diverse cases or discussion of minor themes? | N/A |
